# Case Report: The First Reported Case of Bullous Lichen Planus-Systemic Lupus Erythematosus Overlap Syndrome

**DOI:** 10.3389/fmed.2021.744592

**Published:** 2021-11-04

**Authors:** Yi Liu, Xuelei Liang, Haixuan Wu, Fenglin Zhuo

**Affiliations:** Department of Dermatology, Beijing Friendship Hospital, Capital Medical University, Beijing, China

**Keywords:** bullous lichen planus, systemic lupus erythematosus, overlap syndrome, case report, alopecia

## Abstract

**Introduction:** Lichen planus/lupus erythematosus overlap syndrome is rarely seen in the clinic and has the characteristic clinical manifestations, histopathology, and immunopathology of lichen planus (LP) and lupus erythematosus (LE). This is the first reported case of bullous lichen planus (BLP)/systemic lupus erythematosus (SLE) overlap syndrome with hair loss as the first symptom.

**Case Presentation:** A 48-year-old female presented with alopecia for half a year, and skin lesions accompanied by itching on her face, trunk, and limbs for 3 months. She had a history suggestive of photosensitivity. Laboratory tests and histopathology were performed for diagnosis. Histopathologic features of the upper arm and back of the hand were consistent with BLP, whereas the scalp lesion indicated LE. Laboratory examination indicated positive for antinuclear antibody (ANA) (1:160), leukopenia, increased urinary protein, decreased C3/C4, and normal BP180. The patient was given glucocorticoid combined with acitretin and immunosuppressive therapy after a definite diagnosis of BLP/SLE overlap syndrome. The lesions of the patient disappeared and some hair had regrown during the two years of follow-up.

**Conclusion:** This is the first reported case of BLP/SLE overlap syndrome which responded well to glucocorticoids, retinoids, and immunosuppressive drugs. Multiple biopsies from characteristic lesions will guide doctors to avoid misdiagnoses and delayed treatment.

## Introduction

Lichen planus/lupus erythematosus overlap syndrome is a rare disease characterized by clinical manifestations, histopathology, and/or immunopathology, and cannot be explained alone by either lichen planus (LP) or lupus erythematosus (LE) (1). The lesions often involve the distal arms, legs, face, and trunk, and are characterized by painful, centrally atrophic, and bluish red to hypopigmented in color, large, and scaly patches (2). At present, its etiology is unknown although it may be related to autoimmunity, genetic factors, and virus infection. In addition, isoniazid, procaine amide, or other drugs can also induce this disease (3). Bullous lichen planus (BLP) is a subtype of LP, presenting with vesicles or bullae on flat papules or normal skin. This is the first report of a case of BLP/SLE.

## Case Report

A 48-year-old female presented with hair loss for half a year, skin lesions, and itching on her face, trunk, and limbs for 3 months. She was admitted to Beijing Friendship Hospital of the Capital Medical University (Beijing, China) in February 2019. Previously, she was diagnosed with folliculitis and eczema with which cetirizine, hydrocortisone, and mupirocin were prescribed but symptoms persisted. The lesions gradually increased accompanied by symmetrical pain of the wrist and knee joints but without swelling, fever, cough, and expectoration. The condition would be worse after sunlight exposure. Prior to onset, she had been in good health and had no history of medical drug use.

**Figure 1:**
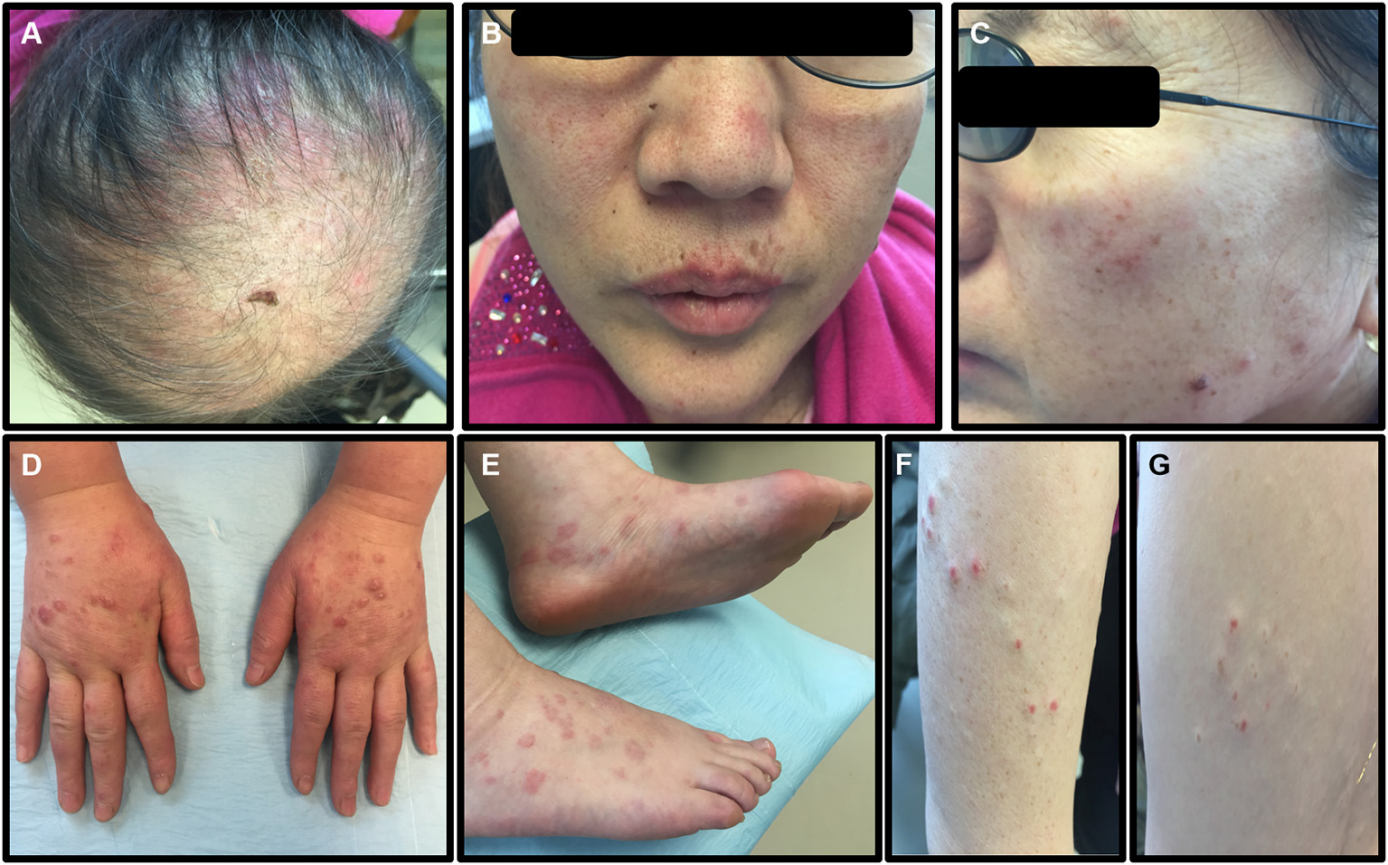
The symptoms of the patient before treatment. **(A)** Large alopecia patches on the top of the scalp. **(B,C)** Erythematous plaques on the scalp and face, with irregular edges and slight scales on the surface. **(D,E)** Violaceous lichenoid papules and blisters can be seen on the back of hands and feet, with clear boundaries. **(F,G)** Papules could be seen on the trunk and arms.

A dermatology examination (Figure 1) showed large alopecia patches on the scalp, erythematous plaques on the scalp and face with irregular edges and slight scales on the surface, violaceous lichenoid papules, and blisters on the back of hands and feet with clear boundaries, and papules could be seen on the trunk and arms. The lesions did not involve the mucosa and are without superficial lymphadenopathy and leg edema.

**Figure 2:**
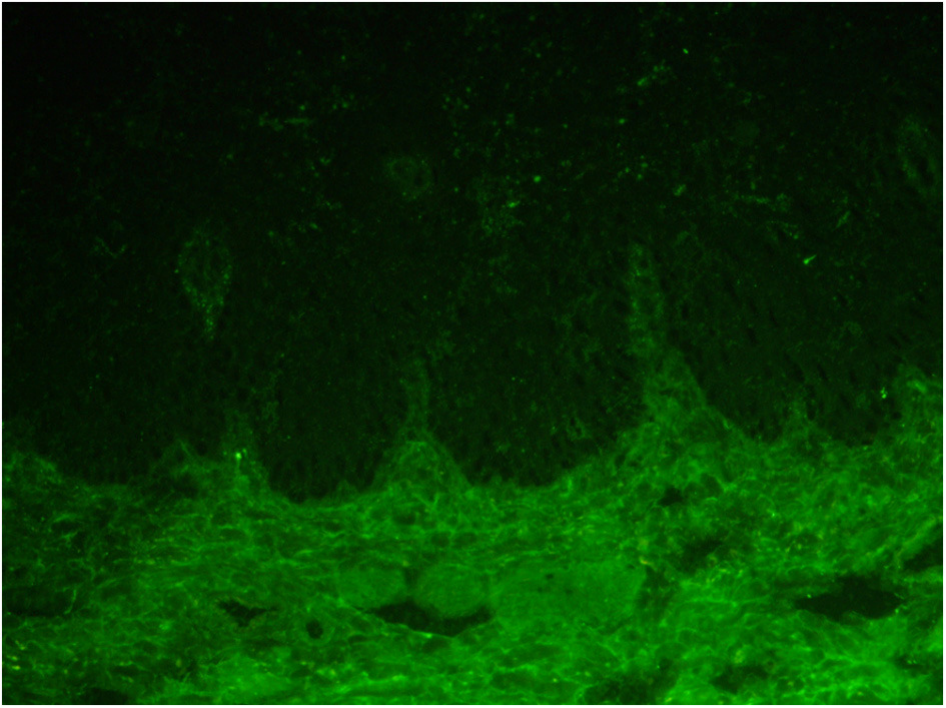
Indirect immunofluorescence of the frozen serum of the patient for IgG, IgA, and C3 was all negative.

Laboratory tests for blood showed WBC 3.31 × 10^9^, RBC 3.7 × 10^12^, HGB 110 g/L. Urine routine was negative with 24-h urine protein quantitation at 248 mg (normal value: 28–141 mg) and ANA: 1:160 (homogeneous spot), 1:80 (cytoplasm), dsDNA (-); C3 37 mg/dl (normal value: 90–180 mg/dl); C4 2.67 mg/dl (normal value: 10–40 mg/dl); IgG 2,040 mg/dl; ESR 58 mm/h; and BP180 was 8 RU/ml (normal value <20 RU/ml). Indirect immunofluorescent assay for IgG, IgA, and C3 were negative (Figure 2). There were no abnormalities in liver and kidney function.

**Figure 3:**
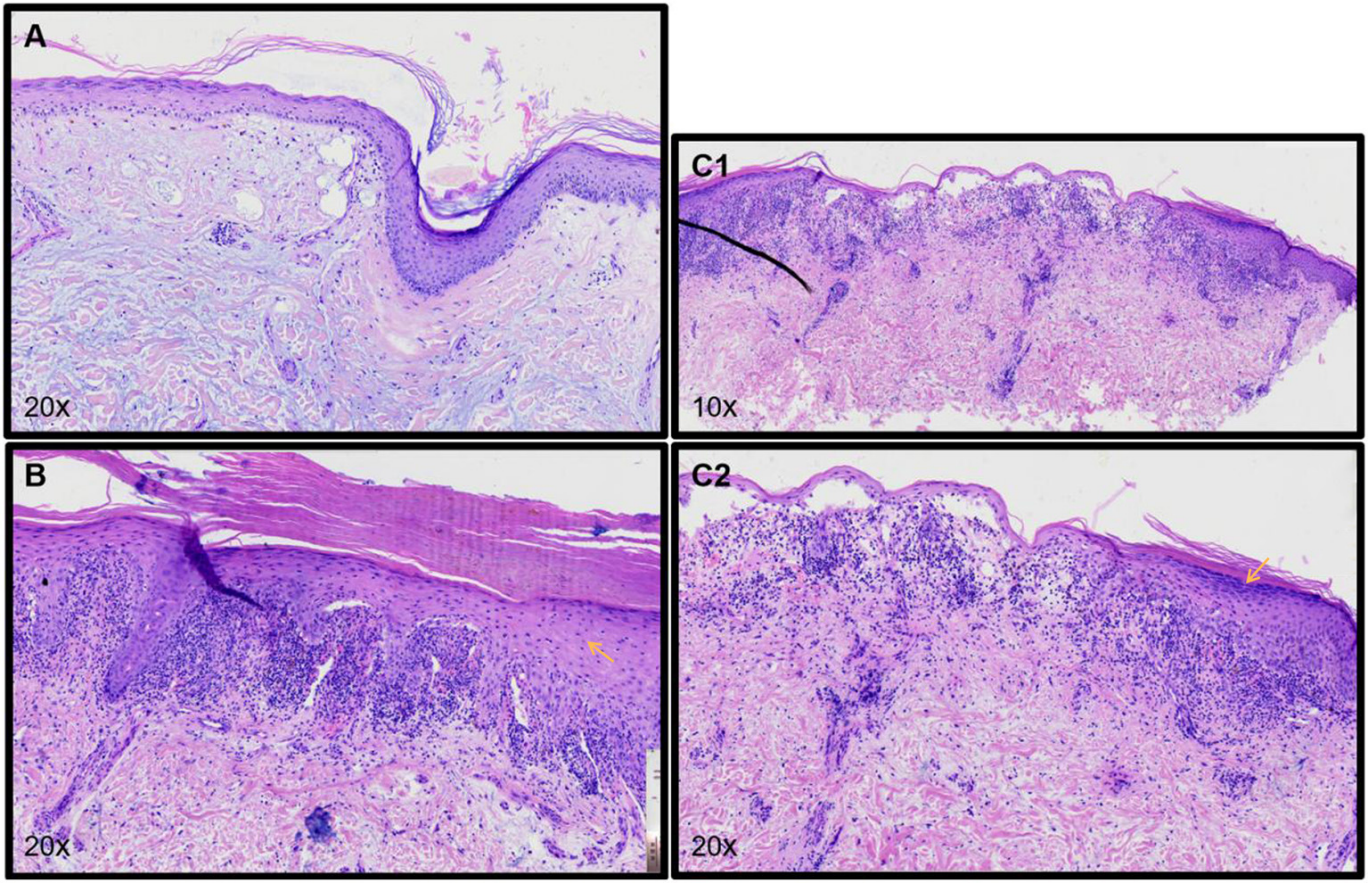
**(A)** Pathology of the scalp shows atrophy of the epidermis, hair follicle angle plug, liquefaction, and degeneration of basal cells, low infiltration of lymphocytes around the superficial dermis vessels, reduction of appendages, and deposition of a large amount of mucoprotein in the dermis. **(B)** Pathology of the upper arm shows hyperkeratosis of the epidermis, wedge-shaped thickening of the granular layer, liquefaction of basal cells, zonal infiltration of lymphocytes in the superficial dermis, and formation of epidermal fissures. **(C1,C2)** Pathology of the back of the patient's hand shows hyperkeratosis of the epidermis, wedge-shaped thickening of the granular layer, liquefaction of basal cells, zonal infiltration of lymphocytes in the superficial dermis, and subepidermal blisters.

Pathological examination (Figure 3) of the scalp showed epidermal atrophy, hair follicle angle plug, liquefaction, and degeneration of basal cells, infiltration around the superficial dermis vessels by a low number of lymphocytes, reduction of appendages, and deposition of a large amount of mucoprotein in the dermis. The scalp pathology and laboratory tests of the patient were in accord with SLE. Pathological examination of the upper arm demonstrated epidermal hyperkeratosis, wedge-shaped thickening of the granular layer, liquefaction of basal cells, zonal infiltration of lymphocytes in the superficial dermis, and formation of epidermal fissures. Pathological examination of the back of the hand showed epidermal hyperkeratosis, wedge-shaped thickening of the granular layer, liquefaction of basal cells, zonal infiltration of lymphocytes in the superficial dermis, and subepidermal blisters. Pathological examination of the upper arm, hand, and back supported the diagnosis of bullous lichen planus. Combined with the history of the patient, skin lesions, laboratory results, and pathological findings, a diagnosis of bullous lichen planus-systemic lupus erythematosus (LP/LE) overlap syndrome was determined.

**Figure 4:**
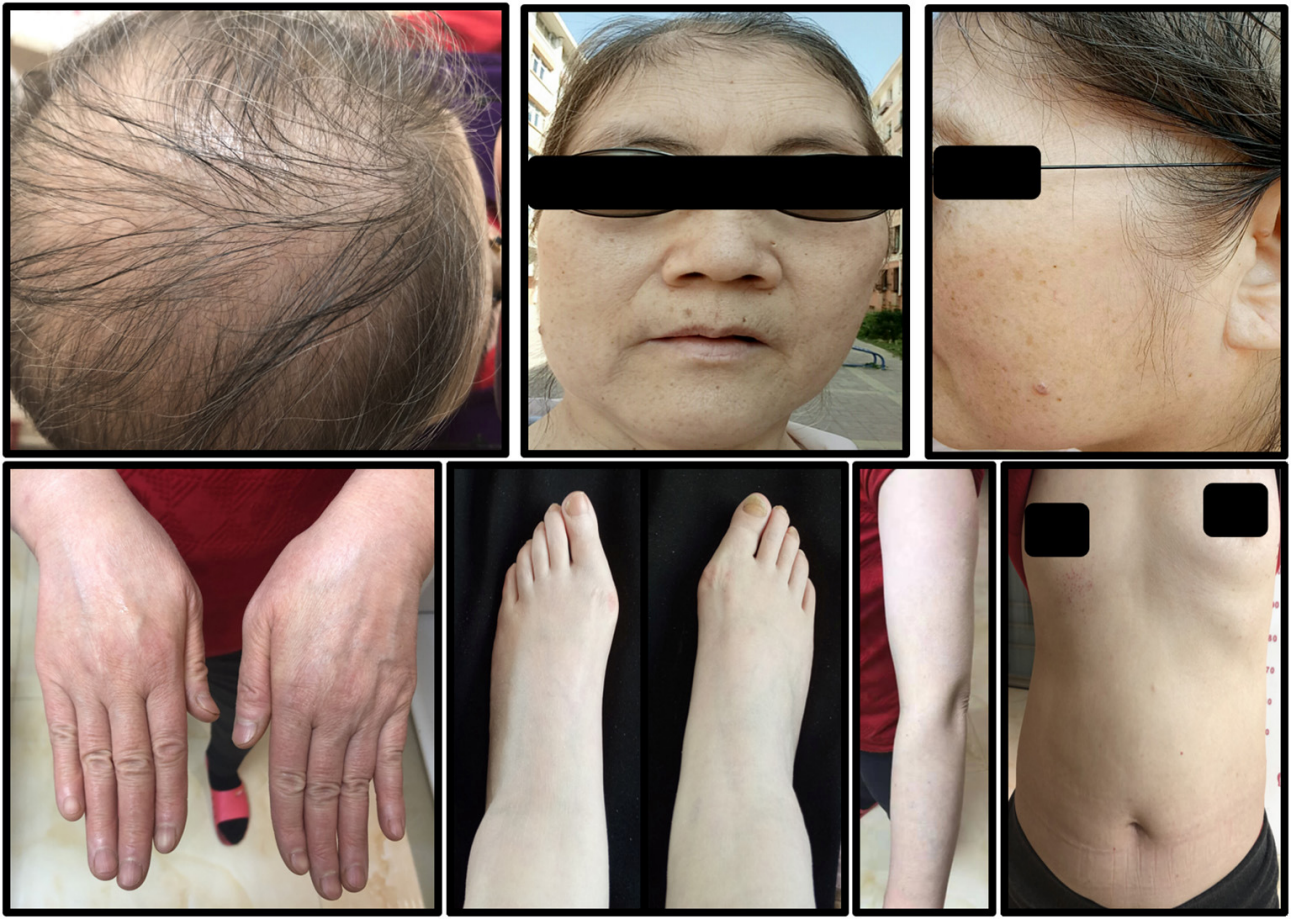
The symptoms of the patient after 2 years of treatment. A small amount of hair had grown and all skin lesions had disappeared.

The patient was given hydroxychloroquine sulfate (200 mg, po, bid), prednisone acetate tablets (40 mg, po, qd), topical 0.05% halometasone cream two times a day for her body, and 0.1% tacrolimus cream two times a day for her face. The symptoms showed significant improvement after 2 weeks. Under these conditions, prednisone acetate was decreased to 20 mg daily, acitretin 30 mg daily, and oral methotrexate 10 mg weekly. About 3 weeks later, the hair of the patient had stopped falling out, the joint pain had disappeared, and partial lesions had regressed. The dosages were gradually reduced according to her symptoms. Upon recent follow-up, her symptoms are stable (Figure 4), her skin lesions have disappeared, and there has been some hair regrowth. Her laboratory test results were normal. Currently, the patient has been prescribed hydroxychloroquine sulfate 200 mg daily, acitretin 10 mg daily, and oral methotrexate 2.5 mg weekly.

## Discussion

To date, ~50 cases of LP/LE overlap syndrome have been reported, most of whom are between 25 and 45 years old, with a predominance of women (4). There is controversy about LP/LE overlap syndrome should be an independent diagnosis specifically for the coexistence of LE and LP as it has been reported that patients have separate lesions consistent with each disease. Alternatively, at least one case report showed real overlap in a single lesion (5). Overlap syndrome is characterized by a mixture of clinical and histopathological features of LE and LP. Direct immunofluorescence shows subepidermal spherical deposits of IgM or IgG or granular deposition of immunoglobulin and complement along the dermal-epidermal junction (lupus zone test) (6). To the best of our knowledge, this is the first reported case of BLP/SLE.

Bullous lichen planus is a rare variant of lichen planus. The formation of blisters in BLP might be related to the severe liquefactive degeneration of basal layer cells. According to the 2019 European League Against Rheumatism/American College of Rheumatology (EULAR/ACR) criteria (7), the ANA (1:160) of the current patient and her total score was 16, including chronic cutaneous lupus (four scores), non-scar alopecia (two scores), non-deformed arthritis, or poly-arthritic pain (four scores), WBC < 4 × 10^9^/L (two scores), and decreased C3 and C4 (four scores), indicating a diagnosis of SLE.

In addition, the diagnosis of the current patient should be differentiated from bullous systemic lupus erythematosus (BSLE) and lichen planus pemphigus (LPP). BLSE is a rare blistering disease, occurred in patients with systemic lupus erythematosus, predominantly in young women. Its clinical characteristics are tension blisters or bullae located on erythema or normal skin, mainly in the trunk, upper limbs, and face (8). Histologically, it showed perivasculitis in the whole layer of the dermis, forming subepidermal blisters within neutrophils infiltrating. Direct immunofluorescence showed linear or granular deposition of IgG, IgA, IgM, and/or C3 in the basement membrane (9). In the current cases, the histological feature around both sides of blisters is hypergranulosis, irregular acanthosis (refer to yellow arrows in Figures 3B,C2). Meanwhile, only superficial dermal vasculitis without neutrophil or leukocyte fragmentation vasculitis was found. Although direct immunofluorescence of skin lesions in this patient is lacking, the histological manifestation met the diagnosis of BLP. Therefore, BSLE was not considered.

Lichen planus pemphigus is a rare and unique autoimmune disease. It is characterized by subepidermal blisters in both lesion and non-lesion LP areas, mainly involving the limbs. The diagnosis depends on clinical, histopathological, and serological results (10). Its histological features are similar to both simple lichen planus and bullous pemphigoid (BP) with the infiltration of most eosinophils and, sometimes, obvious eosinophilic sponge edema. Direct immunofluorescence revealed anti-basement membrane antibodies. In most cases, ELISA showed positive anti BP180 antibody (>20 RU/ml) (11–13). For this patient, no eosinophils infiltration was found in her skin histological examination, the supplementary BP180 was negative (8 RU/ml) and indirect immunofluorescence assay of the frozen serum of the patient for IgG, IgA, and C3 were all negative, which were not consistent with LPP.

Lichen planus/lupus erythematosus overlap syndrome has no standard treatment. However, some patients are resistant to glucocorticoids. Successful treatment of bullous lichen planus with acitretin monotherapy was reported in 2016 (14). In the current case, the patient was resistant to prednisone acetate. The symptoms of the patient were controlled well after adding acitretin and immunosuppressive medicines. Effective treatments for LP/LE overlap syndrome include glucocorticoids combined with hydroxychloroquine, acitretin, thalidomide, cyclosporin, methotrexate, local tacrolimus, and corticosteroids (15–17).

Bullous lichen planus/systemic lupus erythematosus overlap syndrome has the characteristics of BLP and SLE regarding its clinical manifestations, histopathology, and immunopathology. Skin lesions in different areas have specific characteristics that can be confirmed by skin pathology examination. Therefore, clinicians should take multiple samples from different areas containing characteristic lesions to prevent misdiagnosis and delayed treatment.

## Conclusion

This is the first case of BLP/SLE overlap syndrome. The patient recovered well after receiving glucocorticoids in combination with immunosuppressive therapy. Clinicians should take multiple biopsies from the characteristic lesions.

## Data Availability Statement

The original contributions presented in the study are included in the article/Supplementary Material, further inquiries can be directed to the corresponding author.

## Ethics Statement

Written informed consent was obtained from the individual(s) for the publication of any potentially identifiable images or data included in this article.

## Author Contributions

FZ diagnosis and treatment of patients and put forward ideas. YL consulted literature and wrote articles. HW followed up patients and collected photos. XL arranged the pathological pictures. All authors contributed to the article and approved the submitted version.

## Funding

This work was supported by grants from the National Natural Science Foundation of China (No. 81301385).

## Conflict of Interest

The authors declare that the research was conducted in the absence of any commercial or financial relationships that could be construed as a potential conflict of interest.

## Publisher's Note

All claims expressed in this article are solely those of the authors and do not necessarily represent those of their affiliated organizations, or those of the publisher, the editors and the reviewers. Any product that may be evaluated in this article, or claim that may be made by its manufacturer, is not guaranteed or endorsed by the publisher.

## References

[1] Nagao K Chen KR. A case of lupus erythematosus/lichen planus overlap syndrome. J Dermatol. (2006) 33:187–90. 10.1111/j.1346-8138.2006.00043.x 16620224

[2] Vázquez-López F Manjón-Haces JA Maldonado-Seral C Raya-Aguado C Pérez-Oliva N. Dermoscopic features of plaque psoriasis and lichen planus: new observations. Dermatology. (2003) 207:151–6. 10.1159/000071785 12920364

[3] Inalöz HS Chowdhury MM Motley RJ. Lupus erythematosus/lichen planus overlap syndrome with scarring alopecia. J Eur Acad Dermatol Venereol. (2001) 15:171–4. 10.1046/j.1468-3083.2001.00223.x 11495530

[4] Patil P Nayak C Tambe S Das D. Lupus erythematosus-lichen planus overlap syndrome in an HIV-infected individual. Int J STD AIDS. (2016) 27:1117–22. 10.1177/0956462415618109 26582481

[5] Gillian W Michael P. Update on lichen planus and its clinical variants. Int J Womens Dermatol. (2015) 1:140–9. 10.1016/j.ijwd.2015.04.001 28491978 PMC5418875

[6] Sarah S Marjon V Usha A. Seven-year itch: a perplexing case of lichen planus-lupus erythematosus overlap syndrome. Dermatol Online J. (2018) 24:12. 10.5070/D3249041418 30677835

[7] Assan F Seror R Mariette X Nocturne G. New 2019 SLE EULAR/ACR classification criteria are valuable for distinguishing patients with SLE from patients with pSS. Ann Rheum Disundefined. (2019) 80:e122. 10.1136/annrheumdis-2019-216222 31501139

[8] de Risi-Pugliese T Cohen Aubart F Haroche J Moguelet P Grootenboer MS Mathian A. Clinical, histological, immunological presentations and outcomes of bullous systemic lupus erythematosus: 10 New cases and a literature review of 118 cases. Semin Arthritis Rheum. (2018) 48:83–9. 10.1016/j.semarthrit.2017.11.003 29191376

[9] Contestable James J Edhegard Kim D Meyerle Jon H. Bullous systemic lupus erythematosus: a review and update to diagnosis and treatment. Am J Clin Dermatol. (2014) 15:517–24. 10.1007/s40257-014-0098-0 25358414

[10] Matos-Pires E Campos S Lencastre A João A Mendes-Bastos P. Lichen planus pemphigoides. J Dtsch Dermatol Ges. (2018) 16:335–7. 10.1111/ddg.13434_g 29465781

[11] Skaria M Salomon D Jaunin F Friedli A Saurat JH Borradori L. IgG autoantibodies from a lichen planus pemphigoides patient recognize the NC16A domain of the bullous pemphigoid antigen 180. Dermatology. (1999) 199:253–5. 10.1159/000018257 10592407

[12] Sekiya A Kodera M Yamaoka T Iwata Y Usuda T Ohzono A. A case of lichen planus pemphigoides with autoantibodies to the NC16a and C-terminal domains of BP180 and to desmoglein-1. Br J Dermatol. (2014) 171:1230–5. 10.1111/bjd.13097 24813536

[13] Archer CB Cronin E Smith NP. Diagnosis of lichen planus pemphigoides in the absence of bullae on normal-appearing skin. Clin Exp Dermatol. (1992) 17:433–6. 10.1111/j.1365-2230.1992.tb00253.x 1486711

[14] Rallis E Liakopoulou A Christodoulopoulos C Katoulis A. Successful treatment of bullous lichen planus with acitretin mo-notherapy. Review of treatment options for bullous lichen planus and case report. J Dermatol Case Rep. (2016) 10:62–4. 10.3315/jdcr.2016.1235 28435476 PMC5392245

[15] Lospinoso DJ Fernelius C Edhegard KD Finger DR Arora NS. Lupus erythematosus/lichen planus overlap syndrome: successful treatment with acitretin. Lupus. (2013) 22:851–4. 10.1177/0961203313492243 23761099

[16] Demirci GT Altunay IK Sarikaya S Sakiz D. Lupus erythematosus and lichen planus overlap syndrome: a case report with a rapid response to topical corticosteroid therapy. Dermatol Rep. (2011) 3:e48. 10.4081/dr.2011.e48 25386300 PMC4211510

[17] Zhang L Au S Aronson IK. Successful long-term thalidomide therapy for discoid lupus erythematosus-lichen planus overlap syndrome. Dermatol Online J. (2014) 20:13030/qt73r7492v. 10.5070/D32010024267 25526013

